# Sagittal septum duplication of bladder and duplication of posterior urethra combined with congenital megacolon: a case report and literature review

**DOI:** 10.1186/s12894-023-01395-3

**Published:** 2024-01-03

**Authors:** Shuying Luo, Junwei Liu, Peng Su, Hong Yu, Bangguo Li

**Affiliations:** 1 Department of Radiology, Medical Imaging Center of Guizhou Province, Affiliated Hospital of Zunyi Medical University, Zunyi, 563003 China; 2 Department of Urology, Affiliated Hospital of Zunyi Medical University, Zunyi, 563003 China

**Keywords:** Duplication of the bladder, Computed tomography, Magnetic resonance imaging, Case report

## Abstract

**Background:**

Duplication of the bladder with duplication of the posterior urethra is a relatively rare congenital malformation. Cases of sagittal septum duplication of the bladder with duplication of the posterior urethra have rarely been reported. Furthermore, the combination thereof with congenital megacolon is rare.

**Case presentation:**

A 21-year-old male was admitted to our hospital because of frequent urination for two months. He presented to another hospital first with frequent urination and underwent computed tomography (CT) and testicular biopsy. Anti-inflammatory therapy was administered by the doctor to the patient. For further diagnosis and treatment, the patient went to the outpatient department in our hospital on June 6, 2022. After admission, the patient underwent ultrasound, CT, MRI, cystoscopy, and other related examinations and tests. The examination results suggested that the patient had duplication of the bladder with duplication of the posterior urethra. In addition, the patient’s mother reported that he had suffered from long-term constipation with abdominal distension before the age of 5 years. At the time, he was admitted to the local hospital and was diagnosed with congenital megacolon based on the relevant examinations. After the patient was diagnosed with duplication of bladder and urethra, the doctor recommended surgical treatment to the patient. However, he considered that he only had frequent urination symptoms, and chose conservative treatment rather than to undergo surgical treatment. Thus, the doctor prescribed anti-inflammatory treatment. Four months later, the patient reported that frequent urination symptoms persisted, and was also considering fertility-related problems. The outpatient follow-up will be continued.

**Conclusions:**

In this article, we summarize the imaging findings of duplication of the bladder with duplication of the posterior urethra and propose the advantages and disadvantages of each type of imaging examination. We also review the relevant literature on cases of bladders with duplication of the posterior urethra. The related differential diagnosis is summarized, and the significance of guiding clinical treatment and diagnosis is discussed.

## Background

Duplication of the bladder is a relatively rare congenital malformation [1]. There are about 100 cases of duplication of bladder reported worldwide, which are usually associated with genital and distal gastrointestinal duplication, mainly seen in men [2–4]. Duplication of the bladder can be divided into complete duplication and incomplete duplication. The incomplete duplication of the bladder refers to two half-bladders that are not completely separated and are drawn through the same urethra. The complete duplication of the bladder is the presence of two independent bladders, with normal mucosa and muscle layer, separated by a peritoneal fold, each bladder discharged through a separate urethra [5]. There are two types of complete bladder duplication, sagittal and coronal, depending on the axis of the septum [6]. Sagittal septum duplication of the bladder is the most common type [2]. Effmann [7] divided duplication of the urethra into three classifications: Effmann type I: incomplete and blind-end urethral duplication; Effmann type II: complete urethral duplication of one or two openings; Effmann type III: complete or incomplete urethral duplication with bladder duplication or other duplication. Duplication of the bladder is usually diagnosed in infants but is occasionally found in adults [8]. In this case report, we describe a rare case of sagittal septum duplication of the bladder with duplication of the posterior urethra (Effmann type III), combined with congenital megacolon. The patient can grow up without bladder and urethral reconstruction surgery. To the best of our knowledge, there are only 3 cases of sagittal septum duplication of the bladder with duplication of the posterior urethra were reported. Furthermore, a combination thereof with congenital megacolon is even more rare.

We summarize the imaging findings of duplication of the bladder with duplication of the posterior urethra, which is helpful to improve the attention of clinicians and radiologists to this disease, and further make accurate diagnosis and reasonable treatment plan. We present the following article in accordance with the CARE reporting checklist.

## Case presentation

A 21-year-old male was admitted to our hospital because of frequent urination lasting 2 months. When the patient developed symptoms of frequent urination, he presented to another hospital first and underwent some routine tests at admission. Urinalysis revealed 81 white blood cells/ul, normal biochemical indicators of renal function. No sperm was seen on semen analysis; thus, a further testicular biopsy was performed. This revealed a few seminiferous tubules in the testicular tissue, and spermatogenic cells were seen in the lumen, but mature sperm cells were not visible. Because the patient’s urinalysis suggested the presence of a urinary tract infection, anti-inflammatory therapy was administered. When his symptoms subsequently resolved, he asked to be discharged from the hospital.

Nevertheless, frequent urination recurred, and for further diagnosis and treatment, the patient visited the outpatient department of our hospital on June 6, 2022. On physical examination, the patient was found to have an old surgical incision scar, approximately 12 cm in length, on the left side of the abdomen, with no abnormalities of the anus. Routine urinalysis of the patient: 8 white blood cells/ul. In addition, the patient’s mother reported that he had suffered from long-term constipation with abdominal distension before the age of 5 years. At the time, he was admitted to the local hospital and was diagnosed with congenital megacolon based on the relevant examinations. After communication between the attending doctor and the patient’s family, the patient was advised to undergo immediate surgical treatment. The patient underwent resection of the above malformation. The patient’s family members have no similar clinical manifestations and malformations. Given the long interval, we were unable to obtain relevant clinical evidence and specific treatment procedures used for the patient.

**Fig. 1:**
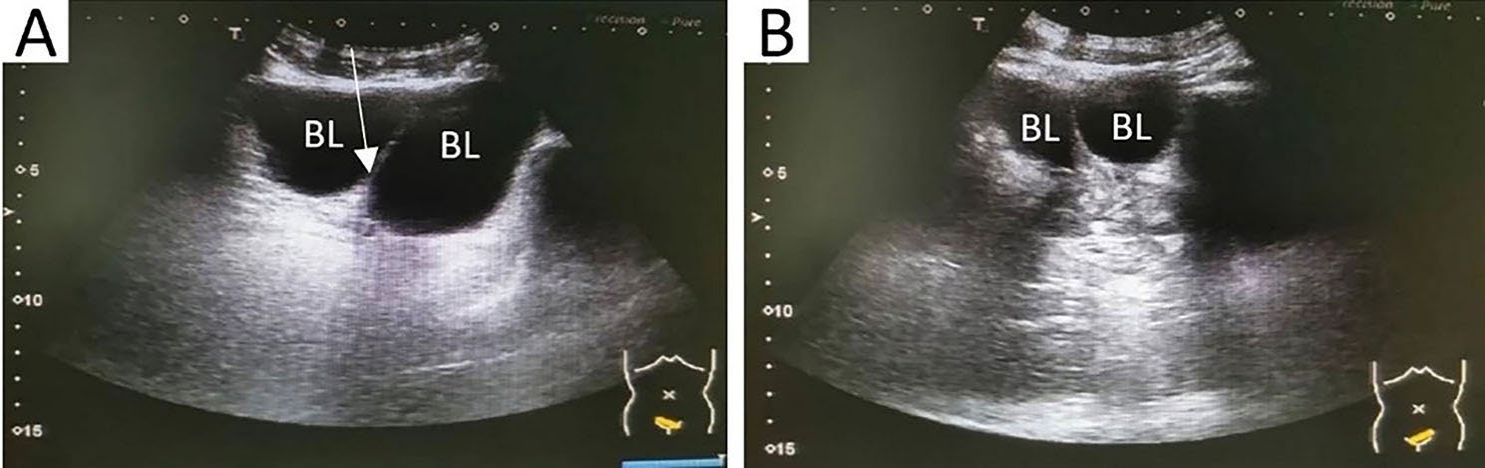
Urinary ultrasound: the left and right bladders (BL) were not connected, which were separated by a septum (white arrow)

**Fig. 2:**
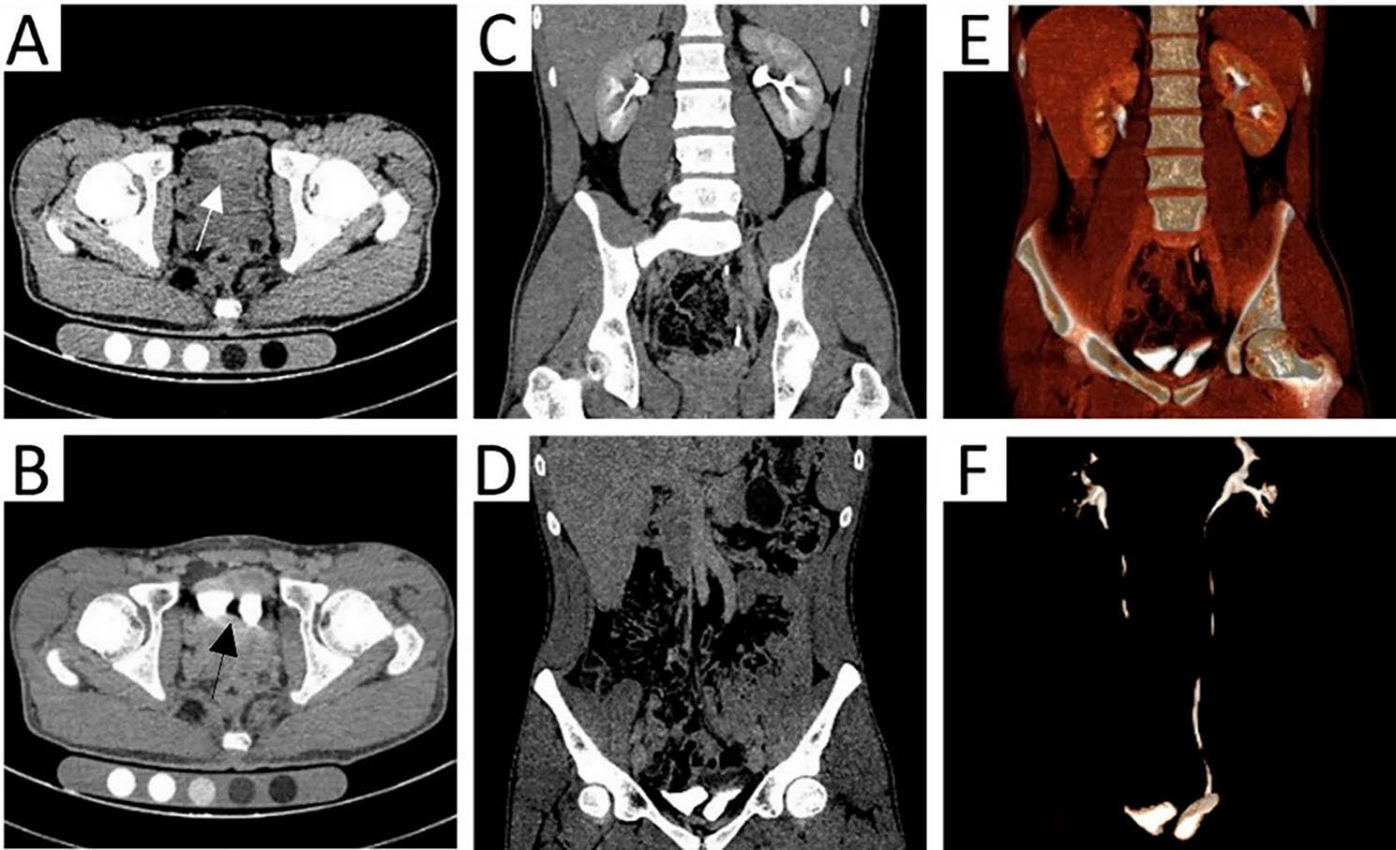
Urinary CT: cross-sectional images: a separation of seven millimeters (white and black arrow) in the bladder **(A**-**B)**, there are two contrasts filled chambers in the pelvic cavity. MIP coronal view: the left and right kidneys were connected to each side of an independent ureter, and the two bladder cavities were side by side **(C**-**E)**. VR: the entire urinary system can be seen. The left and right kidneys and a single ureter connect the left and right bladders **(F)**

**Fig. 3:**
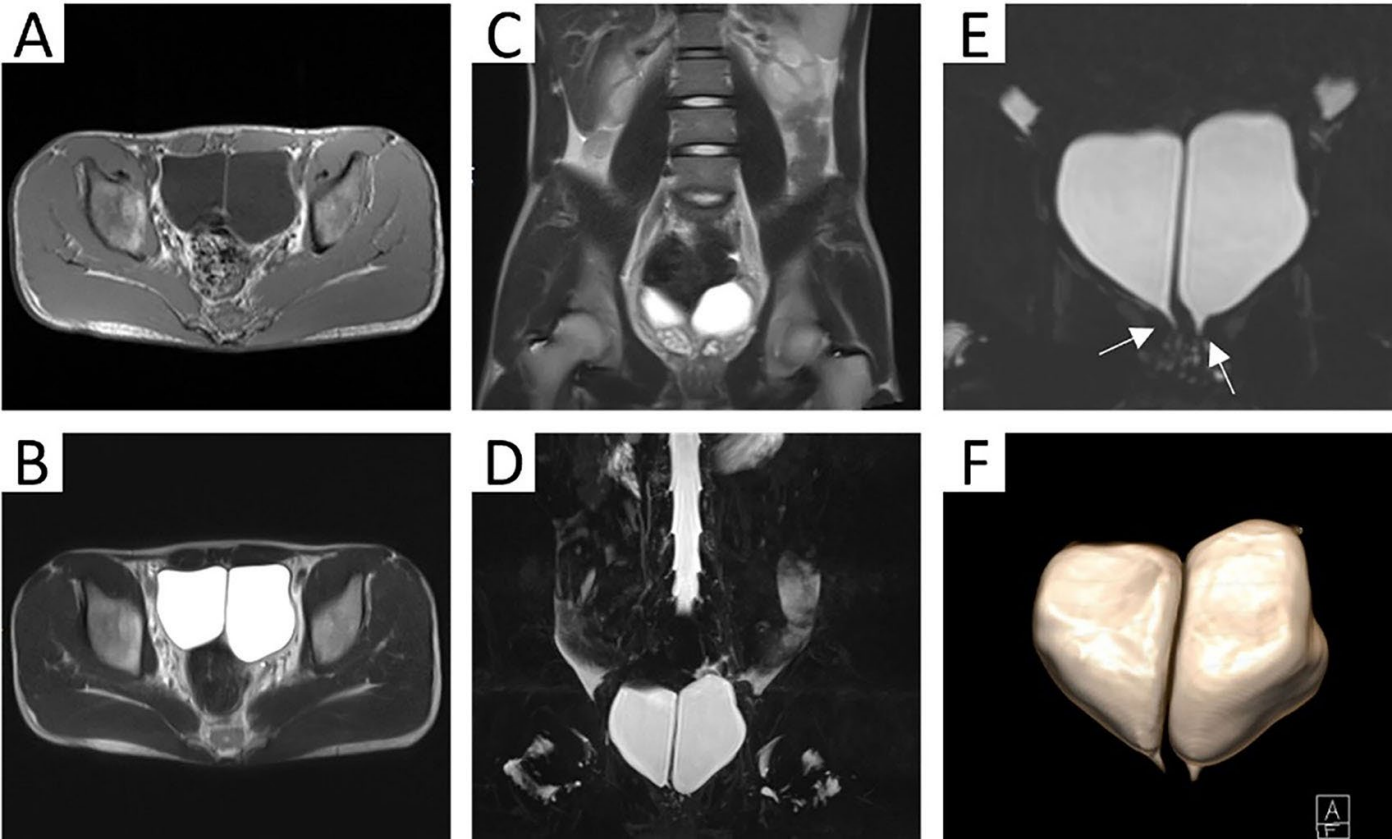
MRI: T1WI, T2WI cross-section and coronal plane: two chambers in the pelvic arranged left and right, the two chambers were separated by a sagittal septum, and septum shape and signal were similar to the bladder wall **(A**-**C)**. MRU: two bladders were connected to the bilateral posterior urethra respectively **(D**-**E)**. 3D: two half-bladders and two posterior urethras **(F)**

At our hospital, an ultrasound examination showed a hyperechoic strip within the dark area of the bladder, which was connected to the posterior wall, and two urethral lines could be seen in the prostate (Fig. 1). Bladder separation or a double bladder was considered. Simultaneously, a plain computed tomography (CT) scan revealed a separation of about 7 mm in the bladder. A delayed enhancement scan displayed that two cavities were filled by the contrast agents in the pelvic cavity, the right half was slightly smaller and the left half was slightly larger (Fig. 2A and B). Maximum intensity projection (MIP) showed that the left and right bladders were connected to the two kidneys through a single ureter on each side (Fig. 2C and D). Volume rendering (VR) presented the entire collection system, showing a single ureter on each side connecting to the left and right bladder cavities, but the urethra was not shown (Fig. 2E and F). The patient also underwent magnetic resonance imaging (MRI): the T1- and T2-weighted images showed a sagittal septum between the left bladder and right bladder, with a thickness and signal similar to those of a normal bladder wall (Fig. 3A and C). Magnetic resonance urography (MRU) revealed two bladders that were separately connected to two urethras (Fig. 3D and F).

**Fig. 4:**
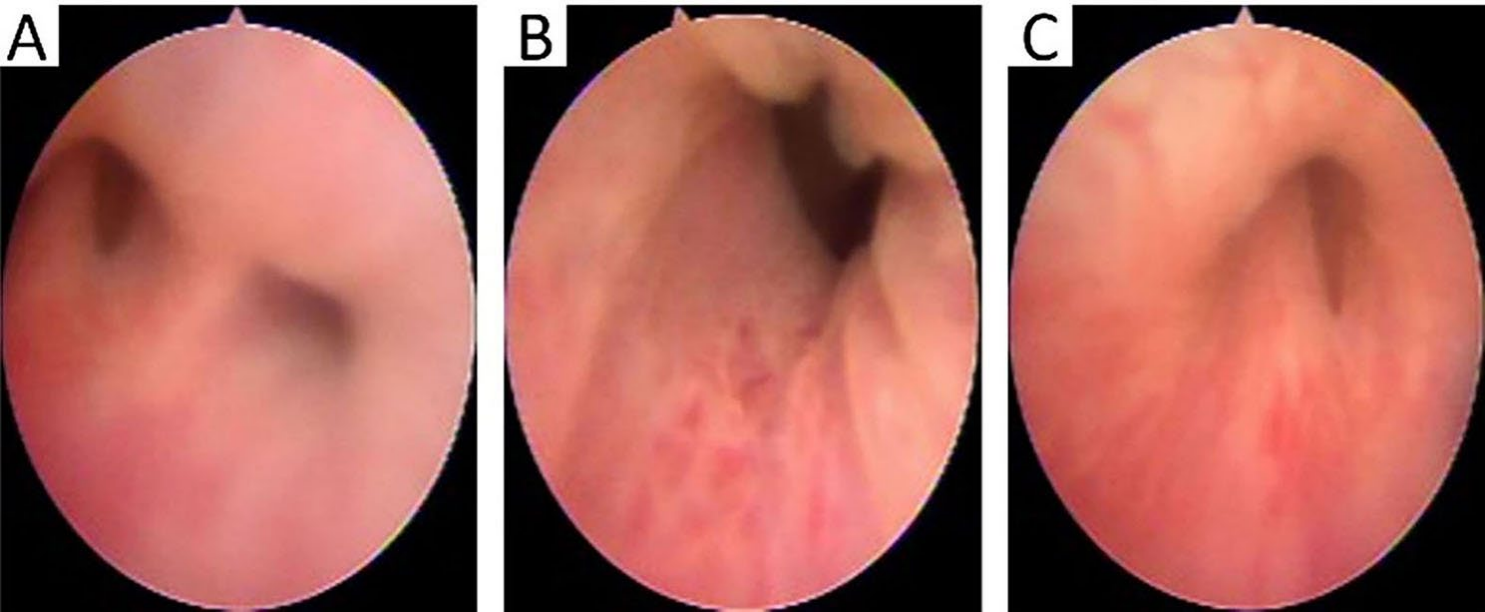
Cystoscopy: the left and right urethras were seen in the membranes part of the urethra **(A)**, the left posterior urethra was irregular in shape, the mucosa was not smooth, and there was no verumontanum structure **(B)**, the right posterior urethra’s verumontanum structure was visible **(C)**

Cystoscopy was performed to clarify the anatomical structure of the malformation (Fig. 4). Two urethras were seen from the membrane part of the urethra. Each urethra entered the left and right incoherent bladders. The diagnosis of sagittal septum duplication of the bladder combined with a duplication of the posterior urethra was confirmed by cystoscopy. Cystoscopy also displayed that a verumontanum was present in the right urethra, but not in the left urethra. Based on combined cystoscopy, CT, and MRI examination, we speculated that the patient’s two bladders could normally discharge urine to the left and right urethras. Our cystoscopy results together with the previous testicular biopsy findings suggested that the patient may have azoospermia (verumontanum obstruction).

After the patient was diagnosed with duplication of bladder and urethra, the doctor recommended surgical treatment to the patient. However, he considered that he only had frequent urination symptoms, and chose conservative treatment rather than to undergo surgical treatment. Thus, the doctor prescribed anti-inflammatory treatment. Four months later, the patient reported that frequent urination symptoms persisted, and was also considering fertility-related problems. The outpatient follow-up will be continued.

## Discussion

### Literature review

**Fig. 5:**
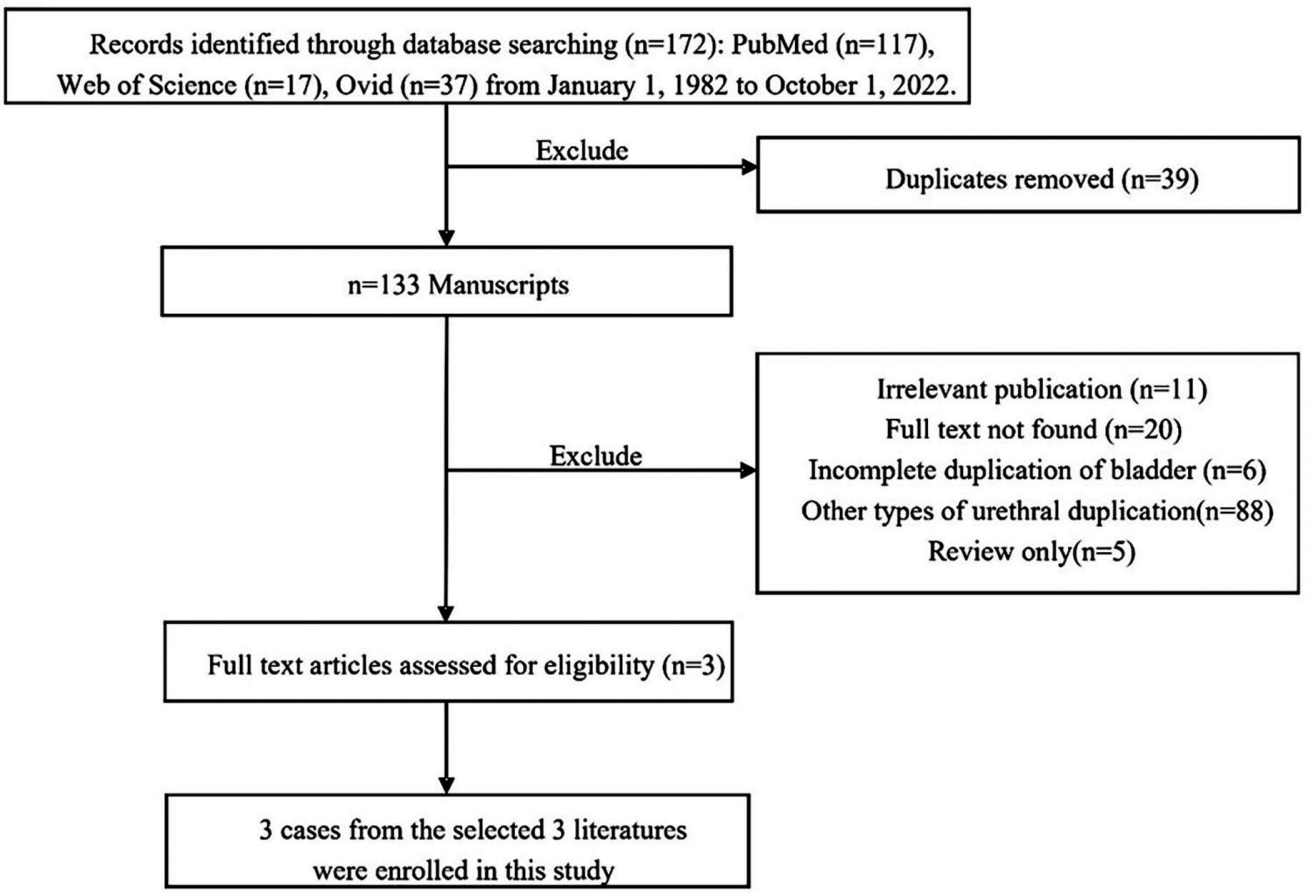
The flow chart of the literature screening process for sagittal bladder duplications and duplication of posterior urethra

To perform the review of the literature, relevant articles in English were extensively searched for the PubMed, Web of Science, Ovid data-base. The period of research was between January 1, 1982, and October 1, 2022. The keywords used for the search were “duplication of bladder”, “double bladder”, “double urethra”, “duplication of urethra”. These words were used individually “OR” with the Boolean operator “AND”. A total of three articles involving three cases were included in the analysis. The flow chart of the literature screening process is set out in Fig. 5. For each case, the data were collected for the first author, year of publication, patient’s age/sex, bladder duplication type, bladder duplication direction, Effmann’s classification, other associated anomalies, and treatment (Table 1).

**Table 1:** Reported cases of sagittal bladder duplication and posterior urethra duplication

Case (No.)	Reference	Age/sex	Bladder duplication type	Bladder duplication direction	Effmann’ Classification	Others Associated Anomalies	Treatment
1	G.J. O’SULLIVAN, J et al.1996	1D/M	Complete duplication	Sagittal duplication	Effmann type III	Imperforate Anus, bowel obstruction	Non surgery
2	Anuruddha M. et al. 2008	12Y/M	Complete duplication	Sagittal duplication	Effmann type III	Duplication of Intestinal	Non surgery
3	Alejandra et al. 2017	1D/M	Complete duplication	Sagittal duplication	Effmann type III	Coronal hypospadias	Non surgery

We have reviewed the literature on three previously reported cases of sagittal septum duplication of the bladder combined with duplication of the posterior urethra. All cases were underage males, two newborns, and one 12-year-old. The newborns were identified due to anal atresia. The adolescent was identified due to dysuria, which was consistent with the reported case. Two cases were diagnosed by intravenous urography (IVU), and one case was diagnosed by micturating cystourethrogram. The two urethras were connected to the left and right bladder respectively, and merged into one urethra at the bulb part of the urethra, in the shape of “Y”.

At present, the embryological basis of bladder duplication remains unclear, and the use of a single theory cannot fully explain all types of bladder duplication [9]. According to Abrahamson’s classification, the embryological origin of bladder duplication may be associated with excessive narrowing between the urogenital system and the ventral vesicoureteral cloaca or additional cloacal septa being pressed into the bladder epithelium, causing it to divide [4]. The division of the embryo’s cloaca is a key step in the formation of the digestive and genitourinary systems [10], and relevant abnormalities in the genitourinary systems could be induced by defects in this process. Therefore, we speculate that the congenital megacolon may be related to the duplication of the bladder and urethra. In addition, the embryonic development of urethral duplication is unclear, because different types of malformations may have different stimulating factors, which may be related to the termination of the cloacal membrane, the development of genital nodules, and some dislocation of the urogenital sinus [11].

Duplication of the bladder and urethra requires multiple imaging techniques to observe the structure and function of the urinary system and to determine the degree and type of duplication [12]. Ultrasound can detect various types of bladder duplication malformations in the early stage and is a suitable screening and follow-up method. In addition, ultrasound usually shows related upper urinary tract abnormalities [13]. For sagittal bladder duplication combined with a posterior urethral duplication, intravenous urography (IVU) usually displays two bladders in parallel, separated by a septum containing the muscular layer, or manifests as two complete, independent bladders. The ipsilateral ureter enters the ipsilateral bladder, and the urethra is “Y-” shaped. The length of urethral duplication also can be determined by IVU [11]. Although IVU is helpful for diagnosis, it is invasive and requires radioactivity. The major advantages of MRU are that it is non-invasive and does not involve radioactivity, and can clearly reveal the anatomical structure of the upper urinary tract and exclude the malformation of the upper urinary tract. The drawback of MRU is that it cannot clearly display the anatomical structure of the lower urinary tract. Additionally, the reconstructive images of CT or MRI can provide a diagnosis and classification, with valuable information, as it can determine the malformation size, location, and relationship with adjacent anatomical structures [14]. CT can reveal the septum containing the muscular layer in the bladder. CT and MRI can demonstrate duplication of the bladder and urethra as well as digestive abnormalities [11]. MRI also has high resolution for soft tissue; thus, it can easily detect bladder duplication, its shape, location, surrounding tissue, and some associated abnormalities. In short, CT or MRI can provide additional three-dimensional information of anatomic structures and their relations and can serve as a useful adjunct in complex clinical scenarios [15]. We are able to understand the anatomical structure of the patient’s malformation through the cystoscopy. Based on this patient, cystoscopy, CT, and MRI can clearly and accurately diagnose bladder and urethral duplication.

Duplication of the bladder needs to be differentiated from bladder diverticula, mesenteric cysts, etc. Bladder diverticula can be secondary or congenital [16]. The complete duplication of the bladder can only be congenitally formed. The clinical manifestations of a bladder diverticulum include hematuria, urinary tract infection, urinary retention, malignant tumor, and rarely, rupture and pain [16]. The clinical symptoms of duplication of the bladder are generally similar to those of a bladder diverticulum. No malignant tumors were found in the previous papers on duplication of the bladder. Sheldon and Essig [17] reviewed the relevant literature: there appeared to be no bladder diverticulum with urethral malformation, genital or distal gastrointestinal system duplication. CT reveals that the wall of the bladder diverticulum is thickened and irregular, while thin-walled structures of different sizes connected to the bladder cavity could be seen on both sides and anterior or posterior walls of the bladder. The bladder diverticulum may potentially develop to a neoplasm, and most of surgeons advocate immediate prophylactic resection of the diverticulum [18].

A mesenteric cyst (MC) is defined as a cyst with an epithelial lining that is located in the mesentery [19]. Most of these cysts are congenital and benign lesions. The causes of MC involve ectopic lymphatic tissue development, abdominal trauma, lymphatic inflammatory obstruction, or localized lymph node degeneration. Its clinical symptoms include abdominal mass, abdominal distension, intermittent abdominal pain, loss of appetite, and severe cases that can be accompanied by fever [20]. Duplication of the bladder can show the same clinical symptoms as MC. MC is showed a low-density shadow inside the abdominal cavity on CT scan, which is closely related to the intestinal tract, but when an MC occurs in the pelvic cavity, the boundary between the MC and bladder may be unclear. Due to the similarity of imaging findings and clinical manifestations between an MC and duplication of the bladder, we should distinguish them. Bladder diverticula, duplication of the bladder, and MC can be diagnosed and differentiated by ultrasound, CT, and MRI.

## Conclusions

In conclusion, for those patients diagnosed with a bladder with urethral duplication, we should carefully detect whether other malformations are present. CT and MRI can reveal the morphology and function of the urinary system as a whole, and can clearly show the separation of the bladder and the two semi-bladder cavities. It is also helpful to observe whether any abnormalities in the reproductive and digestive systems exist. Taken together, for patients with a bladder with urethral duplication, making a definite diagnosis through imaging examination as soon as possible, it is of great significance to guide clinical treatment.

## Data Availability

All data generated or analyzed during this study are included in this published article.

## Abbreviations

CT: computed tomography
ARM: Anorectal malformation
MRI: magnetic resonance imaging
US: Ultrasonography
MIP: Maximum intensity projection
VR: Volume rendering
MRU: Magnetic resonance urography
IVU: Intravenous urography
MCUG: Micturating cystourethrogram
MC: Mesenteric cyst
3D: Three-dimensional reconstruction

## References

[1] Delcont M, Guglielmetti LC, Rajbhandari N, Walker J, Wilcox D, Vuille-Dit-Bille RN (2021). Bladder duplication - a case series. Urology.

[2] Kajbafzadeh AM, Amini E, Javan-Farazmand N, Sahebpour AA (2013). Complex genitourinary duplication affecting neurourologic and urodynamic findings: report of a case and review of the literature. J Pediatr Adolesc Gynecol.

[3] Yang Y, Yang W, Wang Q, Duan Y (2020). Detection of incomplete bladder duplication by SPECT/CT. J Nucl Med Technol.

[4] Abrahamson J (1961). Double bladder and related anomalies: clinical and embryological aspects and a case report. Br J Urol.

[5] Coker AM, Allshouse MJ, Koyle MA (2008). Complete duplication of bladder and urethra in a sagittal plane in a male infant: case report and literature review. J Pediatr Urol.

[6] Galvez C, Guevara C, Nassau DE, Gosalbez R, Alam A (2021). Papers presented at the fall 2020 pediatric urologic oncology work group of the societies of pediatric urology meeting bladder duplication in a setting of VACTER association. Urology.

[7] Effmann EL, Lebowitz RL, Colodny AH (1976). Duplication of the urethra. Radiology.

[8] Gyrtrup HJ, Kure HH (1989). Duplication of the upper and lower urinary tract. Br J Urol.

[9] Djordjevic ML, Stanojevic D, Kojovic V, Ducic S, Joksic I, Pavicevic P (2009). Complete bladder duplication with severe urogenital malformations: embryological and clinical aspects. Eur J Pediatr Surg.

[10] Matsumaru D, Murashima A, Fukushima J, Senda S, Matsushita S, Nakagata N (2015). Systematic stereoscopic analyses for cloacal development: the origin of anorectal malformations. Sci Rep.

[11] Berrocal T, López-Pereira P, Arjonilla A, Gutiérrez J (2002). Anomalies of the distal ureter, bladder, and urethra in children: embryologic, radiologic, and pathologic features. Radiographics.

[12] Tamasi S, Nessuno F, D’Arcangelo R, Di Iorio G, Zeccolini M (2023). Bladder duplication in infant girls: role of imaging in two rare cases with variants of a complete sagittal septum. Pediatr Radiol.

[13] Pirinççi N, Geçit İ, Güneş M, Tanık S, Ceylan K (2013). Complete duplication of the bladder and urethra in the coronal plane: case report with review of the literature. Urol Int.

[14] Li M, Zhang L, Xu XJ, Shi Z, Zhao X-M (2019). CT and MRI features of tumors and tumor-like lesions in the abdominal wall. Quant Imaging Med Surg.

[15] Maciejewski C, Rourke K (2015). Imaging of urethral stricture Disease. Transl Androl Urol.

[16] Kim S, Park SH, Kim DY, Yun SJ, Lee OJ, Han HS (2018). Bilateral obstructive uropathy caused by congenital bladder diverticulum presenting as hypertensive retinopathy. J Korean Med Sci.

[17] Sung CW, Chang CC, Chen SY, Tseng WP (2018). Spontaneous rupture of urinary bladder diverticulum with pseudo-acute Renal Failure. Intern Emerg Med.

[18] Sheldon CA, Essig KenA (1994). Congenital bladder diverticulum causing bladder outlet obstruction: case report and review of the literature. Pediatr Surg Int.

[19] Zia-Ul-Miraj M (1999). Congenital bladder diverticulum: a rare cause of bladder outlet obstruction in children. J Urol.

[20] Shabana A, Dholoo F, Nunn R, Hameed W (2020). Case-report: a rare cause of an intra-abdominal mass. Int J Surg Case Rep.

